# Simplified Approach to Detect Dielectric Constant Using a Low-Cost Microfluidic Quarter Mode Substrate-Integrated Waveguide

**DOI:** 10.3390/s20174985

**Published:** 2020-09-02

**Authors:** Ahmed Salim, Muhammad Usman Memon, Heijun Jeong, Sungjoon Lim

**Affiliations:** School of Electrical and Electronics Engineering, College of Engineering, Chung-Ang University, 221, Heukseok-Dong, Dongjak-Gu, Seoul 156-756, Korea; ahmedsalim789@gmail.com (A.S.); musmanm@outlook.com (M.U.M.); jhijun000015@gmail.com (H.J.)

**Keywords:** substrate-integrated waveguide, RF sensor, chemical sensor, dielectric constant detection, calibration

## Abstract

Liquid materials’ characterization using commercial probes and radio frequency techniques is expensive and complex. This study proposes a compact and cost-effective radio frequency sensor system to measure the dielectric constant using a three-material calibration. The simplified approach measures reflection coefficient magnitudes for all four materials rather than the complex values in conventional permittivity detection systems. We employ a sensor module based on a circular substrate-integrated waveguide with measured unloaded quality factor = 910 to ensure measurement reliability. Miniaturized quarter-mode substrate-integrated waveguide resonators are integrated with four microfluidic channels containing three known materials and one unknown analyte. Step-wise measurement and linearity ensures maximum 4% error for the dielectric constant compared with results obtained using a high-performance commercial product.

## 1. Introduction

Chemical sensing devices have been used to measure fluid purity to streamline aqueous solution preparation for a wide range of industrial and scientific applications. Toxic compounds can be easily injected into humans by inhalation or skin absorption. Intentional or accidental methanol poisoning drops blood pH to 0.3, i.e., acidosis, potentially leading to cell injuries and further complications [1]. Thus, unknown chemicals must be accurately identified to safely and properly utilize toxic chemicals during experimentation in clinical and research laboratories.

Various techniques to detect liquid permittivity have been reported. Conventional detectors and analytical devices examine bio-chemical assays to identify liquid concentrations but require relatively large quantities of liquid to fill reservoirs, tubes, tapes, etc. [2,3]. Thus, significant liquid is wasted, which is often precious (blood, cells, etc.) in the case of biomaterial detection. Microfluidic technology is low-cost, easily fabricated, and requires only micro- or nano-liter fluid samples to detect and analyze fluid types [4]. Polydimethylsiloxane (PDMS) is widely used to manufacture microfluidic assemblies and components due to its flexibility, quick processing, easy fabrication, and minimal cost.

Radio frequency (RF)/microwave resonators integrated with microfluidic technology have been widely reported for various sensing applications [5,6]. Chemical permittivities for low-loss materials have been determined using RF resonance-based sensors from measured resonance frequency and quality factor (QF) changes [7]. High QF, i.e., sharp resonance, implies more accurate detection. The technique can be applied to a wide range of liquid characterization [8,9,10,11,12]. In References [13] and [14], they proposed a substrate-integrated waveguide (SIW) cavity to determine liquid complex permittivity and biomass. SIW and quarter-mode SIW (QMSIW) cavity resonators integrated with microfluidic technology have been subsequently used to detect biomaterial and dual chemicals [15,16]. Perturbation theory links mixture concentration and corresponding resonant frequency and QF changes. RF chemical sensors based on perturbation theory have been proposed recently, which rely on metamaterial [17,18,19,20,21] and substrate-integrated waveguide technology [22,23,24,25].

RF resonator-based chemical sensors are compact, low-cost, and easily fabricated. However, despite these advantages, their applications are limited by narrow-band frequencies, construction material physical properties, and other constraints. The RF chemical sensors also comprise only one component of the measurement system, and vector network analyzers (VNAs) are required to measure sensor response in terms of reflection or transmission coefficients. VNA calibration is somewhat time-consuming since users must perform a sequence of tedious tasks (reset, calibration, measurement) for each time measurement, which precludes rapid detection.

The main purpose of this study was to propose a simplified approach to determine the dielectric constant for any unknown liquid, comprising a compact, cost-effective RF sensor system to measure relative permittivity using a three-material calibration. A system design approach to determine the dielectric constant of an unknown liquid already exists, albeit they include some drawbacks. A six port reflectometer (SPR) system to detect biomass complex permittivity is proposed in Reference [26], comprising a half-mode substrate-integrated waveguide (HMSIW) sensor, RF oscillator, four RF power detectors, and 16-bit data acquisition unit (DAQ). We use three materials with known dielectric properties and estimate reflection coefficient magnitudes for all four materials, providing the dielectric constant for the unknown material. The values are always real, rather than complex, which considerably simplifies the algorithm and circuitry. Consequently, only a single RF power detector is required in the proposed system to measure dielectric constant, whereas similar previous sensors employed four RF power detectors [26]. The second concern in this study was achievement of a high QF. The existing RF resonators targeted for chemical sensing or dielectric characterization exhibit QF in the low to medium range (50~500). We propose a 4-port QMSIW resonator to provide high QF and miniaturize the device.

The complete system to detect liquid dielectric constants comprises the proposed 4-port QMSIW sensor, voltage-controlled oscillator (VCO), RF power detector, and microprocessor. Incorporating the proposed 4-port QMSIW significantly reduces the overall device size. The dielectric constant for any unknown liquid can be measured within seconds after loading the analyte. We compared the proposed sensor performance with a commercial product to highlight the proposed approach efficacy.

## 2. Design: Proposed Dielectric Constant Detection System

### 2.1. 4-Port QMSIW Resonator Design 

The SIW comprises a top conductive surface (rectangular or circular patch) and bottom ground. An inductive wall along the metallic patch boundary shorts the conductive surfaces (top and bottom) and can be easily fabricated by inserting metallic vias. High QFs from SIW-based structures have been a particular research focus due to the resulting enhanced sensing capabilities. The SIW offers conventional waveguides, compact and planar shape, and can accommodate other components and/or microfluidic channels. Half-, quarter-, and eighth-mode SIWs have been proposed to reduce SIW size and enhance performance [27,28]. From image theory, in-phase fields occur on opposite sides of the SIW, and each bisection of the symmetric plane reduces original structure size by half (and hence fourth and eighth), although each bisected sector retains a significant portion of the electric field distribution, sufficient for sensing applications.

### 2.2. Modeling

**Figure 1:**
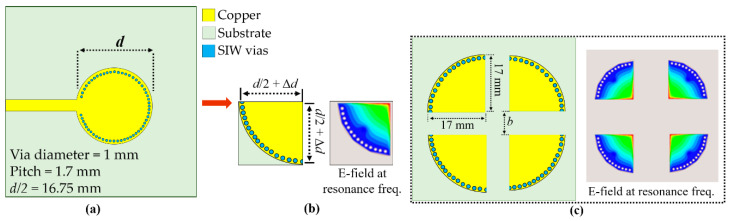
Proposed 4-port quarter-mode substrate-integrated waveguide (QMSIW) cavity resonator developed from the circular full substrate-integrated waveguide (SIW) cavity resonator: (**a**) microstrip line (MSL)-fed circular SIW cavity resonator, (**b**) coaxial-fed QMSIW cavity resonator, and (**c**) 4-port QMSIW resonator and corresponding electric field (E-field) distribution at 4.5 GHz.

Figure 1 shows the proposed 4-port QMSIW based on a circular SIW cavity resonator (4.5 GHz) to achieve high QF [21]. The detailed design, dimensions, and electric field distribution for the microstrip line (MSL)-fed circular SIW cavity have been presented previously [23], and modification from a circular SIW to a 4-port QMSIW is detailed in Figure 1.

Substrate-integrated waveguide structures maintain their radiation characteristics when they are miniaturized or split into equal portions. Figure 1b shows that QMSIW cavity sides are not exact d/2, where d is the diameter of the original circular cavity, because fringe fields are generated from the QMSIW cavity open sides [29]. Hence, we add some small Δd to ensure the structure retains the same dominant mode as the full SIW cavity. Nevertheless, physical size was reduced to ¼ by realizing the QMSIW cavity with side length = d/2 + Δd = 17 mm.

We designed the 4-port QMSIW to enable four microfluidic RF sensors on a single board: one for the test liquid and three for the calibration liquid samples. The 4-port QMSIW sensor uses Rogers Duroid 5880 substrate with dielectric constant ε_r_ = 2.2, loss tangent = 0.0009, and thickness = 1.575 mm. Four identical QMSIW resonators were placed as shown in Figure 1c, requiring little more total space than a single circular SIW.

**Figure 2:**
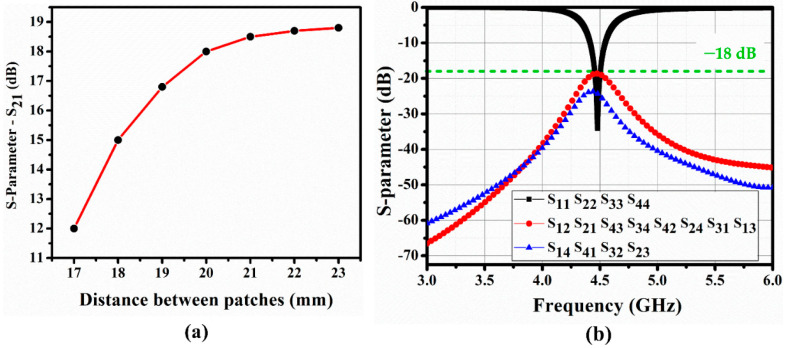
(**a**) Simulated isolation between ports 1 and 2 (S_21_) for different distances, a, between quarter-mode substrate-integrated waveguide (QMSIW) segments (see Figure 1), and (**b**) S-parameters for 4-port QMSIW at b = 20 mm.

The distance b between every 2 QMSIW resonators was decided considering the isolation between each port. A larger b increases the isolation at the cost of an increase in the overall size. Hence, we selected the optimized value of b to reduce the overall size. Figure 2a shows isolation between ports 1 and 2 (|S_21_|) for different values of b, which are derived using the ANSYS high-frequency structure simulator.

Since |S_21_| saturates for b > 20 mm, we chose b = 20 mm, and Figure 2b shows the corresponding S-parameters for the 4-port QMSIW resonator. Isolation between the ports exceeds 18 dB at 4.5 GHz, and return loss at each port = 33 dB at 4.5 GHz.

**Figure 3:**
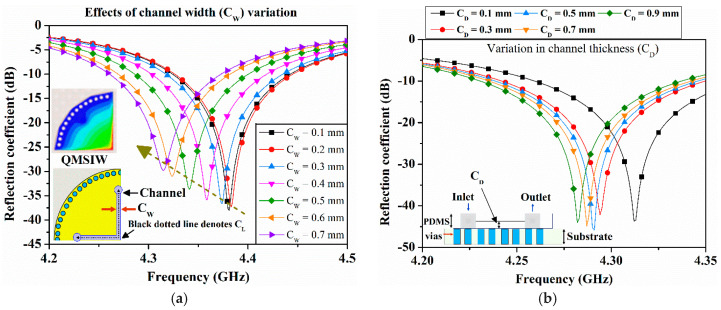
Simulated reflection coefficient of port 1 (S_11_) for a variation in (**a**) the channel width (C_W_) when the first microfluidic channel is filled with diluted ethanol (ε_r_ = 52 and tanδ = 0.35), and (**b**) the channel depth (C_D_) when the first microfluidic channel is filled with diluted ethanol (ε_r_ = 52).

Microfluidic channels are placed over the four QMSIW cavities, employing three channels for internal calibration and the other to sense the unknown liquid. The microfluidic channel used a PDMS substrate with ε_r_ = 2.7, loss tangent = 0.02, and thickness = 1 mm. The length, width, and depth of each microfluidic channel is denoted as C_L_, C_W_, and C_D_, respectively. In SIW designed at fundamental mode (TE_10/01_), the highest E-field resides in the center and in QMSIW, the E-field is aligned along the bisected walls of each QMSIW, as can be seen from Figure 1c. To maximize QMSIW sensor frequency variation for different liquids, microfluidic channels were placed at locations that generated maximum electric field (E-field) [29]. Therefore, C_L_ is aligned with the bisected walls of QMSIW, as can be seen in the inset of Figure 3a. The width of the ethanol-filled channel is varied from 0.1 to 0.7 mm within a 1 mm-thick PDMS, and the resulting S_11_ from port 1 are shown in Figure 3a. An increased volume causes downshift in resonance frequency and as expected, the magnitude of S_11_ decreases. Next, we investigated variations in C_D_ from 0.1 to 0.9 mm with a step size of 0.2 mm, and the resulting reflection coefficients of port 1 are provided in Figure 3b. Considering fluidic flow and fabrication limitations, microfluidic channel width and depth were selected as 0.5 mm.

QMSIW resonance frequency with empty microfluidic channels = 4.55 GHz, which was considered as the reference throughout this study.

**Figure 4:**
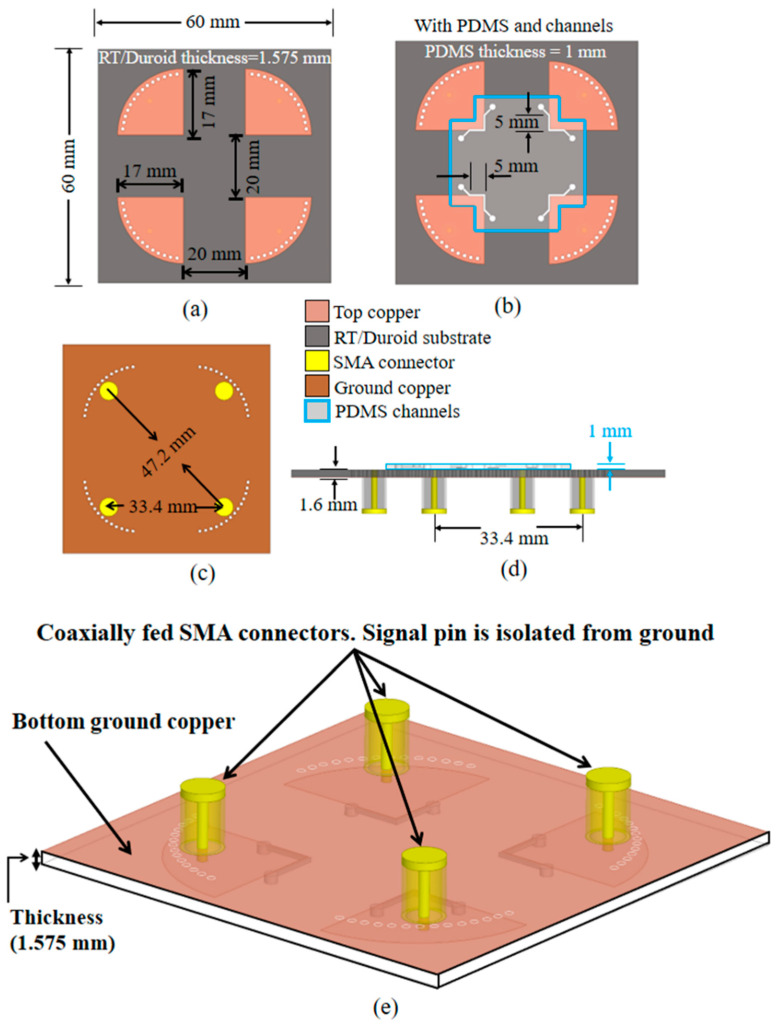
Proposed 4-port quarter mode substrate-integrated waveguide sensor design with physical dimensions: completed structure top view (**a**) without and (**b**) with microfluidic channels, (**c**) bottom view, (**d**) cross-sectional view, and (**e**) bottom view, angled.

Figure 4 shows the final 4-port QMSIW sensor design and physical dimensions. Figure 4a,b shows the completed structure top view without and with microfluidic channels respectively, and Figure 4c,d shows bottom and side views with 4 sub-miniaturized version A (SMA) connectors, which were included in the electromagnetic (EM) simulations.

### 2.3. Establish High Q Proposition

**Figure 5:**
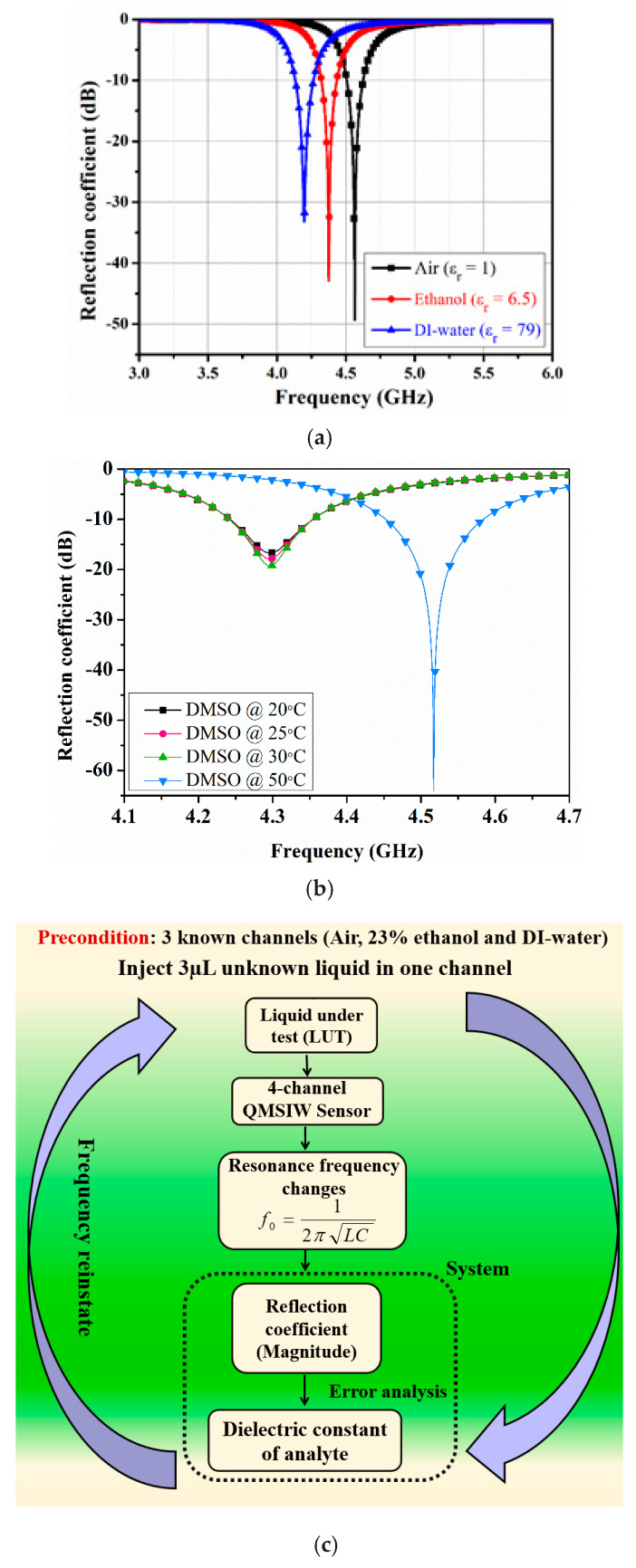
(**a**) Simulated reflection coefficients for the proposed 4-port quarter-mode substrate-integrated waveguide resonator. Dielectric properties were considered at room temperature (20 °C), (**b**) Simulated reflection coefficient of port 1 (S_11_) when the first microfluidic channel is filled with the dimethyl sulfoxide (DMSO) for a range of temperatures. The remaining three ports are kept open. (**c**) Proposed simplified approach.

Figure 5a shows the proposed sensor-simulated return loss for port 1. Port 1 resonates at 4.55 GHz (with 49 dB return loss) when the microfluidic channels are empty, and 4.37 and 4.2 GHz with ethanol and deionized (DI) water, respectively. Ethanol and DI water dielectric properties were simulated as *ε*_r_ = 6.5 and loss tangent = 0.38, and *ε*_r_ = 74.7 (@ 4.2 GHz) and loss tangent = 0.55, respectively [30,31].

To collect reliable data, the intended calibration materials must have precise data at required conditions. Low-cost and easily available materials are preferred. DI water, ethanol, acetone, methanol, benzene, and propanol have been extensively studied at various temperatures and wide frequency range.

The simulated loaded QF was calculated from QF = *f_o_/f*_3*dB*_, where *f_o_*, and *f*_3*dB*_ represent the resonance frequency and 3 dB bandwidth. We estimated 3 dB bandwidth from the S-parameters (Figure 4), and infer

$$\mathrm{air}\;(\mathrm{empty}\;\mathrm{microfluidic}\;\mathrm{channel}):\;\mathrm{QF}=\frac{f_{o}}{f_{3dB}}=\frac{4.55\;\mathrm{GHz}}{(4.564-4.563)\;\mathrm{GHz}}=4550,$$

$$\mathrm{ethanol}:\;\;\mathrm{QF}=\frac{f_{o}}{f_{3dB}}=\frac{4.37\;\mathrm{GHz}}{(4.377-4.374)\;\mathrm{GHz}}=1456,\;\mathrm{and}$$

$$\mathrm{DI}\;\mathrm{water}:\;\;\;\mathrm{QF}=\frac{f_{o}}{f_{3dB}}=\frac{4.20\;\mathrm{GHz}}{(4.202-4.192)\;\mathrm{GHz}}=420$$

The lower QF for ethanol and DI water compared with the empty channel is due to the higher loss tangent for these lossy liquids.

Besides ethanol–water mixtures, the simulations are extended, for instance, temperature effects on dielectric properties is investigated. Dimethyl sulfoxide (DMSO) is a colorless solvent that dissolves both polar and nonpolar compounds and is commonly used in biological experiments and the pharmaceutical industry. A DMSO-filled-channel is simulated for a range of temperatures, whereas the remaining three channels were rendered empty, and results are shown in Figure 5b. From Reference [32], the dielectric properties of DMSO are considered as follows: at 20 °C (ε = 36.57, tanδ = 0.48), at 25 °C (ε = 37.44, tanδ = 0.44), at 30 °C (ε = 38.07, tanδ = 0.41), and at 50 °C (ε = 38.84, tanδ = 0.31).

Figure 5c shows the proposed simplified dielectric constant extraction approach. Three known channels hold air, 23% ethanol, and DI water, and the unknown liquid is injected into the fourth microfluidic channel. Reflection coefficient magnitude is then estimated for each liquid.

**Figure 6:**
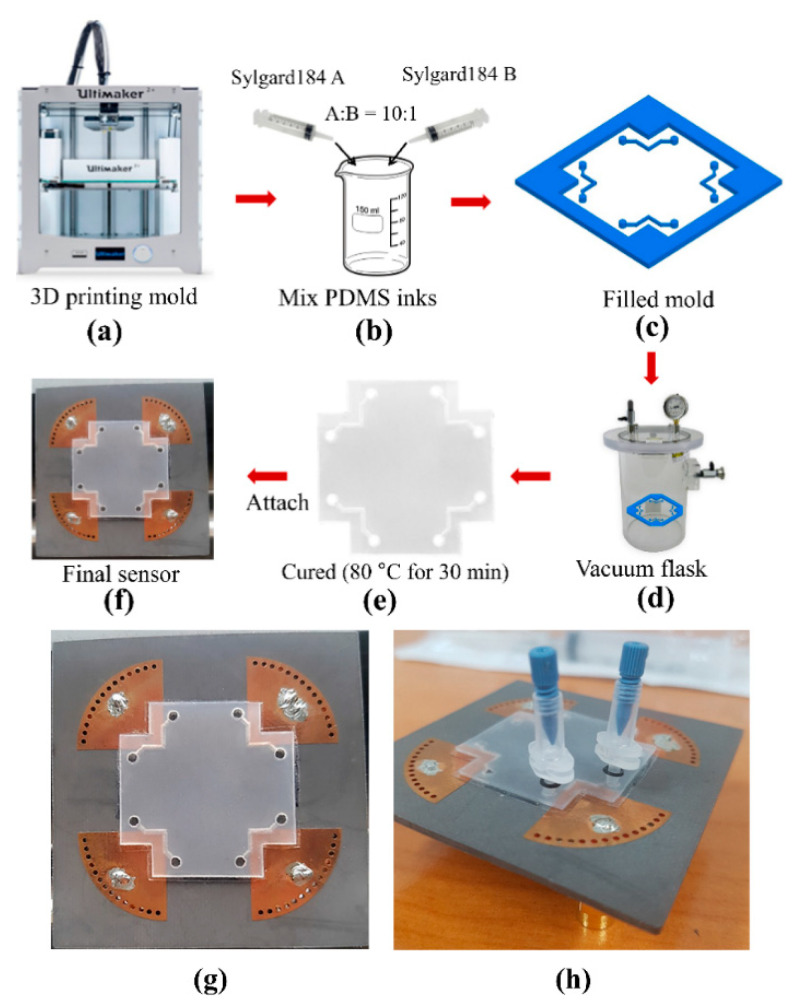
(**a**–**f**) Microfluidic channel fabrication using polydimethylsiloxane (PDMS), (**g**) fabricated 4-port quarter-mode substrate-integrated waveguide resonator prototype top view, and (**h**) nanoport assembly at microfluidic channel inlet and outlet for precise injection and removal of the liquid under test.

Microfluidic channels were fabricated with alignment marks to ensure accurate location. Figure 6 shows the microfluidic channel fabrication process. First, we prepared a mold for the microfluidic channel using a three-dimensional (3D) printer (Ultimaker 2+) (Figure 6a). Two PDMS elastomers (Sylgard 184 A and B) were mixed at 10:1 mass ratio, stirred (Figure 6b), and poured into the 3D mold (Figure 6c) until the internal walls were covered. The filled mold was then placed in a vacuum for 30 min (Figure 6d), separated, and cured for 30 min (Figure 6e). Since the 3D mold dimensions were reliable and accurate, all 4 microfluidic channels in the PDMS had identical dimensions. The PDMS patch with microfluidic channels was attached to the QMSIW resonator using double-sided adhesive (thickness = 0.06 mm; Adhesive Research Company) (Figure 6f). Each microfluidic channel was tested with red ink to ensure they were leak-proof. Figure 6g,h shows the final 4-port circular QMSIW resonator sensor prototype.

## 3. Fabrication and Sensor Measurements

The proposed QMSIW was fabricated on a 1.6 mm Rogers/Duroid 5880 substrate using conventional lithography. A copper coating was placed inside the drilled holes to realize the shorting vias. Each QMSIW was coaxially fed by an SMA connector, with the SMA signal pin only contacting the top conductor and the SMA flange soldered to the ground conductor (Duroid 5880).

**Figure 7:**
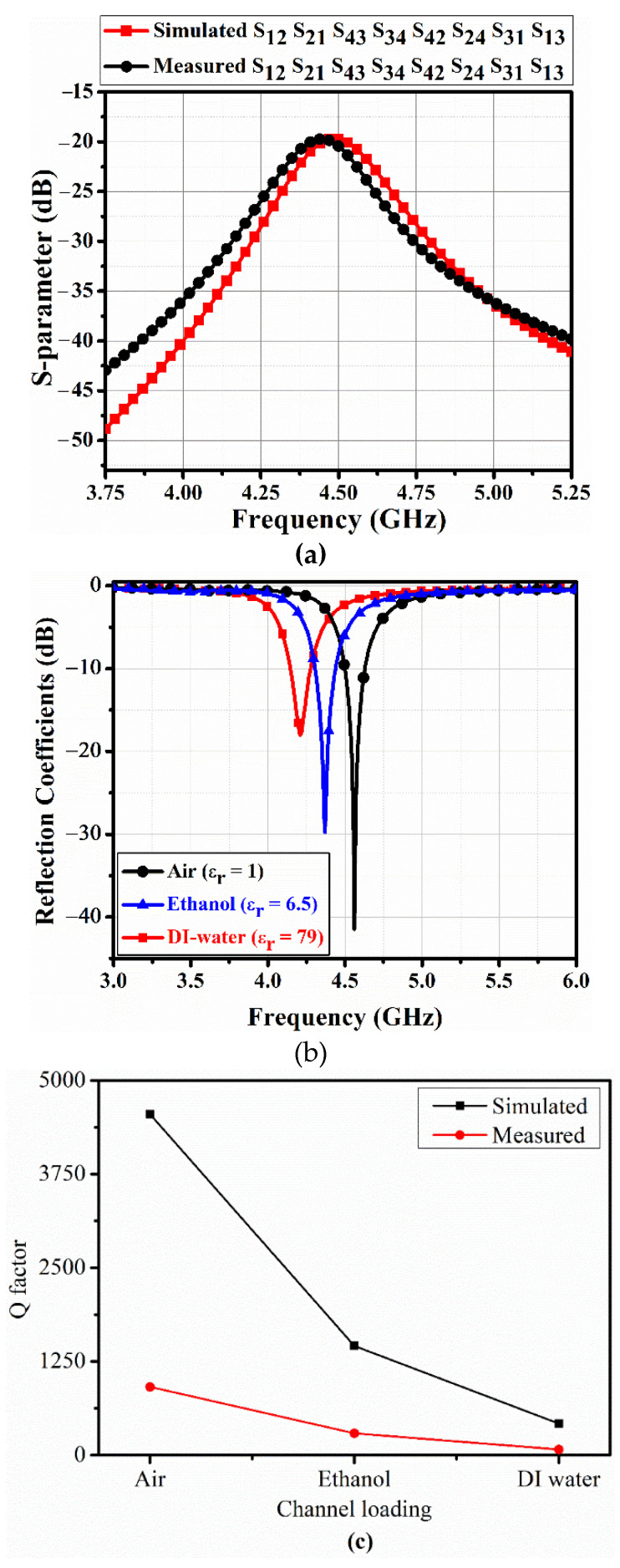
Fabricated 4-port quarter-mode substrate-integrated waveguide resonator (**a**) transmission S-parameters with empty microfluidic channels, (**b**) measured reflection coefficients for port 1 (S_11_), and (**c**) Simulated and measured Q factors (QFs) of the 4-port QMSIW resonator with air, ethanol, and deionized water (DI water).

Figure 7 shows measured S-parameters for a single port of the fabricated 4-port QMSIW sensor using the Anritsu MS2038C network analyzer (Anritsu Corporation, Kanagawa Prefecture, Japan), with an empty microfluidic channel. Isolation between each port > 18 dB with return loss > 40 dB at 4.55 GHz. Figure 7b shows the corresponding port 1 (S_11_) coefficients for air, ethanol, and DI water in the microfluidic channels with corresponding resonances = 4.55, 4.37, and 4.2 GHz, which agreed well with the simulation. Return losses for air, ethanol, and DI water = 41, 30, and 18 dB respectively, at the appropriate resonances. Measured QFs = 910, 291, and 76 respectively, which were approximately 5-fold lower than simulated QFs. This discrepancy in QFs was partially due to error induced by substrate losses and fabrication tolerances, but largely due to differences between simulated and actual PDMS loss tangent. PDMS loss tangent = 0.05 from measured S-parameters, whereas we assumed 0.027 for the EM simulation. Figure 7c shows the simulated and measured QFs for air, ethanol, and DI water materials in the proposed 4-port QMSIW resonator.

## 4. Dielectric Constant Detection System

**Figure 8:**
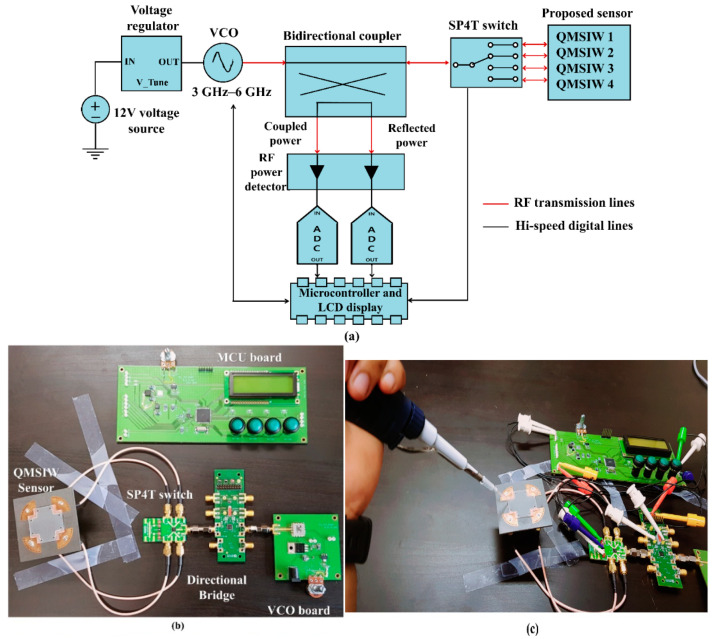
The proposed built-in system to determine unknown dielectric constant: (**a**) block diagram. The radio frequency (RF) power detector measures the power amplitude, and the micro-control unit (MCU) determines resonance frequency corresponding to peak S_11_ amplitude internal algorithm settings, (**b**) prototype circuit modules, and (**c**) measurement setup.

Figure 8a,b shows the proposed dielectric constant detection system block diagram and final prototype, respectively. The system comprises a 4-port QMSIW sensor, voltage-controlled oscillator (VCO) (DCRO316568-5, Synergy Microwave Corporation [33]), bidirectional coupler, RF power detectors (EVAL-ADL5920, Analog Devices Company [34]), analog-to-digital converter (ADC) with micro-control unit (MCU, Microchip Company [35]), and liquid crystal display (LCD, CLCD216-G, COMFILE Technology Company, Sterling, VA, USA).

Figure 8b shows the bidirectional coupler and RF power detectors integrated on the MCU board with the ADC, LCD, and microprocessor. The ADL5920 circuit (Analog Devices company) operated over 9 kHz to 7 GHz with 50 dB at 1 GHz forward power measurement range. The VCO (DCRO316568-5) operating temperature range = −40 to +85 °C and we conducted the experimentation at room temperature (20 °C) [34].

Three known standards (air, 23% ethanol, and DI water) were loaded onto QMSIW ports 1, 2, and 3 respectively, and the test sample was loaded onto port 4 to measure the liquid dielectric constant. Sekar et al. considered test material sample volumes from 50 to 200 µL and observed only slight oscillating variation in extracted dielectric constant [36]. Thus, volume variation has negligible influence on extracted dielectric constant. Channel dimensions = 20 × 0.5 × 0.5 mm (C_L_ × C_W_ × C_D_), hence volume carrying capacity = 5 µL. The fluids (3 µL) were easily injected into each microfluidic channel using a micropipette [37]. Then, the VCO transmitted 4.5 dBm constant RF power sweeping 3–6 GHz to the 4-port QMSIW through the bidirectional coupler, controlling the input frequency sweep by sweeping 0–12 V DC using the microprocessor. Reflected RF power from each QMSIW port was sequentially transmitted to the RF power detector using a single-pole four-throw switch. The RF power detector sensor input power level was rectified to DC and provided root-mean-square (RMS) voltage at its output terminal, which was input to the ADC. The microcontroller unit calibrates the various losses (cable, RF switching, RF power detector) and estimates the reflection coefficient magnitude. Finally, we determine the unknown dielectric constant from the reflection coefficient, as detailed in the following section.

## 5. Dielectric Constant Determination Using Simplified Approach

High QF structures ensure high accuracy liquid property detection [38]. The prototype 4-channel QMSIW sensor system was used to characterize unknown liquid dielectric constants.

We used three calibration materials (*A*, *B*, and *C*) with known reflection coefficients (Γ) and permittivities (*ε*). Inserting unknown reflection coefficients, Γ*_X_*, the test sample complex permittivity (*ε_X_*) can be determined as [39]:

(1) $$\frac{\left(\varepsilon_{X}-\varepsilon_{A})(\varepsilon_{B}-\varepsilon_{C}\right)}{\left(\varepsilon_{X}-\varepsilon_{B})(\varepsilon_{C}-\varepsilon_{A}\right)}=\frac{\left(\Gamma_{X}-\Gamma_{A})(\Gamma_{B}-\Gamma_{C}\right)}{\left(\Gamma_{X}-\Gamma_{B})(\Gamma_{C}-\Gamma_{A}\right)}$$

where subscripts *A*, *B*, and *C* refer to air, 23% ethanol, and DI water respectively, with complex permittivity *ε_A_* = 1, *ε_B_* = 60.24 − j9, and *ε_C_* = 74.7 − j14.99, respectively [31,32,40].

Regarding permittivity, the real permittivity component is always larger than the imaginary component [41]. DI water relative permittivity increases with increasing temperature and decreases with increasing frequency, and the imaginary component increases up to a threshold with increasing temperature and frequency [31]. Therefore, we designed the resonator for approximately 4.5 GHz resonance ranges because DI water permittivity does not change significantly in this range. Thus, errors due to different resonance frequencies are not significant. Since considering only real components provides significantly simpler circuitry to implement permittivity detection using 3-calibration materials, we discarded the imaginary component in Equation (1).

We calculated 1.1% saline complex permittivity from the complex reflection coefficient (Γ) measured by the VNA as *ε*_X_ = 67.2730 − 13.1349j from Equation (1). Since we used the scalar measurement to determine unknown liquid dielectric constants from Γ magnitude, we modified Equation (1) as:

(2) $$\frac{\left(\varepsilon_{rX}-\varepsilon_{rA})(\varepsilon_{rB}-\varepsilon_{r}{}_{C}\right)}{\left(\varepsilon_{rX}-\varepsilon_{rB})(\varepsilon_{r}{}_{C}-\varepsilon_{rA}\right)}=\frac{\left(\left|\Gamma_{X}\right|-\left|\Gamma_{A}\right|)(\left|\Gamma_{B}\right|-\left|\Gamma_{C}\right|\right)}{\left(\left|\Gamma_{X}\right|-\left|\Gamma_{B}\right|)(\left|\Gamma_{C}\right|-\left|\Gamma_{A}\right|\right)}$$

Measured reflection coefficients were |Γ*_A_*| = 0.0079, |Γ*_B_*| = 0.0316, |Γ*_C_*| = 0.125, and |Γ*_X_*| = 0.0392. Hence *ε_X_* = 64.92 from Equation (2), whereas the commercial system returned dielectric constant for 1% saline = 67.5. If we use the complex reflection coefficient, the error is 0.34%. Nevertheless, dielectric constant from the proposed system differs 3.85% from the dielectric constant from the commercial system. Trading off between cost and accuracy, we used the measured reflection coefficient magnitude to enable a low-cost, quick, and portable system, adopting Equation (2) to model the dielectric constant extraction system.

## 6. Results and Discussion

This study utilizes a 3-material calibration technique in a rather simplified way to determine an unknown dielectric constant, in which high QF is achieved using a 4-QMSIW incorporated with a PDMS patch containing four microfluidic channels, with each one aligning on the highest sensitive region of each QMSIW. High QF is the main proposition in this study and we established it in a previous section. Table 1 compares QFs obtained from existing SIW resonators used for chemical sensing.

To demonstrate the performance of our proposed system, we measured relative permittivity for seven liquids: 100%, 70%, 50%, and 25% ethanol, and 0.5%, 1%, and 2% saline using the proposed method and a commercial probe permittivity detection system (Keysight’s N1501A Dielectric Probe Kit) [44]. The commercial probe was employed to provide reference data to validate the proposed system.

The measured dielectric constant error (*E*_1_) can be expressed as:

(3) $$E_{1}=\left|\frac{\varepsilon_{r}-\left|\varepsilon_{Y}\right|}{\left|\varepsilon_{Y}\right|}\right|\times 100\%$$

where *ε_r_* and *ε_Y_* are measured dielectric constants from the proposed and commercial system, respectively. Table 2 summarizes the measured dielectric constants and corresponding error.

The commercial Keysight N1501A system requires a separate network analyzer and computer, whereas the proposed sensor system is self-contained. Thus, the proposed relative permittivity system can accurately measure liquid dielectric constants for much lower cost (~1/500, 0.2%) in a single compact module.

**Figure 9:**
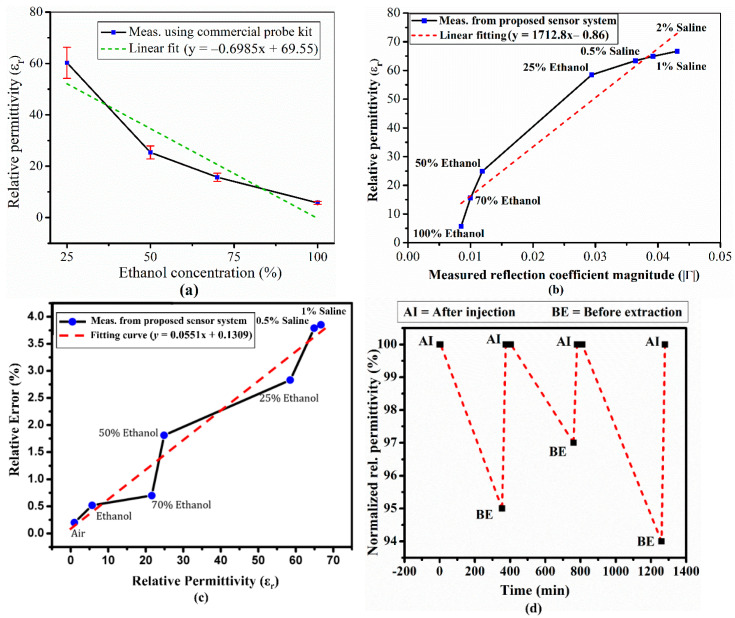
(**a**) Measured relative permittivity for four different ethanol concentrations using the commercial probe kit, (**b**) measured return loss with respect to relative permittivity using the proposed system, (**c**) permittivity relative error for 7 liquids using the proposed system, and (**d**) repeatability/stability for the proposed system (ethanol relative permittivity changed from 5.72 to 5.78 after being left in the microfluidic channels for 5 h).

The linearity of a measuring instrument is analyzed to evaluate reliability and accuracy [45]. In our study, the linear curve fitting model is used to interpret the trend of the measured data. Figure 9 shows the measurement results for samples under test obtained from our proposed detection system and commercial probe kit. To validate the measurement reliability using our proposed detection system, first, the accuracy of reference data is ensured. Figure 9a shows the measured relative permittivity for 25–100% ethanol solutions obtained from commercial probe kit. Despite utilizing a high-performance commercial probe kit, noticeable deviation exists at all measured data points as compared to curve fitting model. Calibration process, solution mixing, and/or averaging of each sample could refine the measurement.

Figure 9b shows measured reflection coefficient magnitude and relative permittivity for each injected liquid. Measured data points deviate at various points and maximum deviation is found for 25% ethanol. Higher permittivity corresponding to Saline solutions (0.5%, 1%, and 2%) closely approached the linear curve. Figure 9c shows relative error with respect to estimated permittivity determined using the proposed system, whereas the “Relative Error (%)” is referred to % Error E_1_, estimated from Equation (3) and shown in Table 2.

Pure ethanol has zero water concentration and the proposed system dielectric constant differs only 0.52%. As water concentration increases in the binary mixture, the increasing imaginary part influences overall permittivity. The increasing DI water concentration increases the imaginary part, and hence discarding that term generates higher error. Therefore, measured dielectric constant corresponding to 0.5%, 1%, and 2% saline solutions exhibit the highest deviations from reference data. We must consider the error due to discarding the imaginary part of permittivity. Consequently, the lower concentration of ethanol (high permittivity/high imaginary part) generates higher error. The proposed approach considers only the real permittivity component, trading between cost and accuracy. The proposed scalar vector network analyzer is considerably cheaper than current commercial VNAs, whereas the VNA was more accurate. Therefore, we investigated the proposed simplified approach using measured reflection coefficient magnitude to provide a low-cost, quick, and portable measurement system.

Ethanol was left (injected) inside the prototype system microfluidic channels for approximately 5 h, with subsequent relative permittivity change from 5.72 to 5.78. Figure 9d shows normalized relative permittivity with respect to time. The relative permittivity change is attributed to shrinking/swollen PDMS when ethanol remains on it for a long time. However, in practice, the proposed detection system takes only a few minutes to inject the three known and one unknown liquids, hence the measurement is quite rapid despite sequentially loading each channel and registering its response. Therefore, there is little or no deviation/error risk during normal operation/processing.

The proposed 6-port reflectometer with four RF power detectors was 1/5 the cost for the commercial single port automatic network analyzer, which could achieve maximum standard error of performance ≈ 1.489% [26]. In contrast, the proposed simplified approach used only a single RF power detector and achieved 4% maximum error compared with the commercial probe. Current products, whether commercial or research focused, are bulky and utilize complex permittivity, hence high equipment and operational costs and tedious calibration requirements.

The proposed simplified detection system is considerably lower cost and more compact compared with the conventional 6-port reflectometer [26], while still providing acceptable accuracy. However, the proposed approach has some limitations, such as discarding imaginary reflection coefficient components introduced a minor error and increasing relative error with increasing relative permittivity.

## 7. Conclusions

This study proposed a compact and cost-effective RF sensor system to detect relative permittivity using a three-material calibration. We designed and fabricated a 4-port QMSIW resonator to provide high QF and miniaturization. Three of the four QMSIW ports use known liquid materials (e.g., air, DI water, and 23% ethanol) to provide automated calibration. The proposed simplified approach measures return loss and reflection coefficient magnitude to calculate the dielectric constant for a test liquid.

We fabricated a prototype system to experimentally demonstrate the proposed concept, incorporating the 4-port QMSIW resonator, VCO, RF detector, and MCU. Relative permittivities for 100%, 70%, 50%, and 25% ethanol, and 0.5%, 1%, and 2% saline were measured using the prototype and compared with results from a commercial dielectric probe (Keysight’s N1501A). The proposed sensor system differed from the commercial device by 0.52–3.89%, with <1% difference for test liquid dielectric constant <20. Therefore, the proposed sensor system can successfully measure liquid dielectric constants for very low cost in a single compact module without requiring a VNA. The proposed simplified approach works well for liquids with low dielectric constants.

For practical applications, overall size could be further reduced by integrating and realizing all components on a single chip, which would greatly enhance the potential application scope.

## Figures and Tables

**Table 1:** QF comparison obtained from recent SIW resonators used for chemical sensing.

Reference	Technology	Simulated Q	Measured Q
This work	4-QMSIW	4550	910
[23]	EMSIW	51	N/S
[24]	SIW	39.12	N/S
[25]	Circular SIW	1080	N/S
[42]	SIW	356	334
[43]	* CSRR-loaded QMSIW	262	N/S

N/S: Not specified, * Complementary split ring resonator (CSRR).

**Table 2:** Measured dielectric constants using the proposed and commercial system.

Sample	Solution			Proposed System		Commercial Kit	Evaluation
	DI Water (ml)	Ethanol (ml)	NaCl (g)	|Γ_X_|	Dielectric Constant ε_r_	Reference [39] ε_y_	% Error E_1_
100% Ethanol	0	350	0	0.0085	5.7	5.75	0.52
70% Ethanol	105	245	0	0.010	15.6	15.71	0.7
50% Ethanol	175	175	0	0.0119	24.9	25.36	1.81
25% Ethanol	225	125	0	0.0294	58.5	60.24	2.88
0.5% Saline	400	0	2	0.0364	63.4	65.9	3.79
1% Saline	400	0	4	0.0392	64.9	67.5	3.85
2% Saline	400	0	8	0.0431	66.7	69.4	3.89
